# First Discovery of Cholesterol-Lowering Activity of Parthenolide as NPC1L1 Inhibitor

**DOI:** 10.3390/molecules27196270

**Published:** 2022-09-23

**Authors:** Wenjing Liu, Bing Liang, Jun Zeng, Jingsen Meng, Lingyu Shi, Shanbo Yang, Jing Chang, Chao Wang, Xiaokun Hu, Xufu Wang, Na Han, Chenghui Lu, Jiao Li, Congcong Wang, Huanting Li, Renshuai Zhang, Dongming Xing

**Affiliations:** 1 Cancer Institute, The Affiliated Hospital of Qingdao University, School of Basic Medicine of Qingdao University, Qingdao 266071, China; 2 Qingdao Cancer Institute, Qingdao 266071, China; 3 Department of Neurosurgery, The Affiliated Hospital of Qingdao University, Qingdao 266071, China; 4 Department of Nuclear Medicine, The Affiliated Hospital of Qingdao University, Qingdao 266071, China; 5 Interventional Medicine Center, The Affiliated Hospital of Qingdao University, Qingdao 266071, China; 6 School of Life Sciences, Tsinghua University, Beijing 100190, China

**Keywords:** cholesterol-lowering ability, PTL, NPC1L1, hypercholesterolemia, drug screening

## Abstract

Elevated cholesterol significantly increases the risk of developing atherosclerosis and coronary heart disease. The key to treating hypercholesterolemia is lowering plasma cholesterol levels. There have been no studies on the cholesterol-lowering potential of parthenolide (PTL), a naturally occurring small molecule from Tanacetum parthenium. Here, we first put forth PTL’s cholesterol-lowering ability to inhibit cellular uptake of cholesterol in a dose-dependent manner. Its performance was on par with the positive control drug, ezetimibe. Niemann–Pick C1 Like-1 (NPC1L1) has been identified as a potential therapeutic target for hypercholesterolemia. The interaction of PTL with NPC1L1 could be explained by the results of molecular docking and filipin staining further reinforces this hypothesis. Furthermore, PTL reduced the expression of NPC1L1 in HepG2 cells in a concentration-dependent manner, which suggests that PTL functions as a potential NPC1L1 inhibitor with therapeutic potential for hypercholesterolemia.

## 1. Introduction

Cardiovascular diseases are the leading cause of morbidity and mortality worldwide [1]. Hypercholesterolemia is a significant risk factor for cardiovascular disease. The key to treating hypercholesterolemia is lowering plasma cholesterol levels. Statins, ezetimibe, and evolocumab are currently the three main medications used to reduce cholesterol. By preventing the rate-limiting step in cholesterol synthesis, which is mediated by 3-hydroxy-3-methylglutaryl-CoA reductase (HMG-CoA reductase), statin medication reduces hepatic cholesterol production. In addition, it reduces low-density lipoprotein (LDL) by 20 to 45 percent [2]. By specifically blocking the NPC1L1 protein at the jejunal brush barrier [3], NPC1L1 inhibitors prevent intestinal cholesterol absorption and offer a vital adjunctive method for treating hypercholesterolemia [4]. Evolocumab is a monoclonal anti-proprotein convertase subtilisin/kexin type 9 (PCSK9) antibody that prevents serum PCSK9 from binding to the hepatic low-density lipoprotein receptor and lessens low-density lipoprotein receptor degradation, enhancing the clearance of serum-circulating low-density lipoprotein cholesterol [5]. However, some patients are still unable to reach their ideal LDL targets after statin therapy, and up to 15% of patients cannot endure the potential side effects of high-dose statins [6]. A small percentage of patients who received ezetimibe orally also suffered from side effects such as myalgia, increased blood creatine kinase, increased transaminases, and thrombocytopenia [7].

Natural products are an important source for screening new medications. Natural products benefit from precise therapeutic actions and have few negative effects [8]. Numerous investigations are being conducted to uncover natural phytochemical substances that can decrease cholesterol [9,10]. The development of new cholesterol-lowering drugs could be accomplished using natural products.

PTL is a sesquiterpene lactone extracted from the leaves of Tanacetum parthenium. PTL’s anti-cancer abilities were first demonstrated in 1973 [11]. PTL has shown anti-cancer activity against many tumor types such as colorectal cancer [12], melanoma [13], pancreatic cancer [14], prostate cancer [15], cervical cancer [16], kidney cancer [17], etc. In addition, it exhibits potent anti-inflammatory properties and is widely used worldwide to treat rheumatoid arthritis and prevent migraine headaches [18]. PTL has been demonstrated to be a powerful NF-B activation inhibitor and is able to reduce the expression of pro-inflammatory cytokines in both cultivated cells and experimental animals [19,20]. Furthermore, Monica L. Guzman et al. [21] reported that PTL induces apoptosis in human acute myeloid leukemia stem cells and progenitor cells. The vital benefits of PTL are its anti-inflammatory, anti-proliferative, and anti-cancer properties while not affecting normal cells [22]. During adipocyte differentiation, it has been noted that PTL reduces lipid accumulation by increasing Nrf2/Keap1 signaling [23]. There is currently no evidence that PTL affects how cholesterol is absorbed. In a preliminary analysis using the NBD approach, we demonstrated that PTL could reduce cholesterol uptake in Caco-2 and HepG2 cells. For the uptake of exogenous cholesterol, NPC1L1 is a crucial target. Through computer simulations, we discovered that PTL could connect to NPC1L1’s cholesterol-binding site. We consequently proposed the hypothesis that PTL may inhibit NPC1L1 and reduce cellular cholesterol absorption. Subsequent experiments further reinforced this hypothesis. PTL inhibited NPC1L1’s ability to transport cholesterol competitively and affected the expression of NPC1L1.

## 2. Results

### 2.1. PTL Inhibits Cholesterol Absorption in Caco-2 and HepG2 Cells

**Figure 1:**
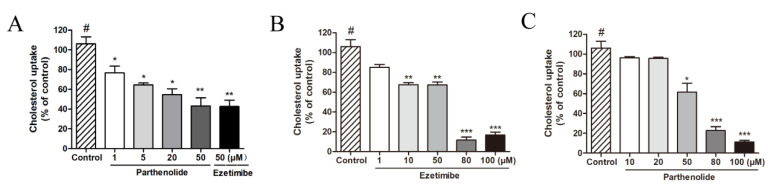
(**A**) Inhibitory effect of PTL on cholesterol absorption in Caco-2 cells. Caco-2 cells were pretreated with PTL at different concentrations for 4 h and then incubated with NBD-cholesterol for 1 h. (**B**) Inhibitory effect of ezetimibe on cholesterol absorption in HepG2 cells. HepG2 cells were pretreated with ezetimibe at different concentrations for 4 h and then incubated with NBD-cholesterol for 1 h. (**C**) Inhibitory effect of PTL on cholesterol absorption in HepG2 cells. HepG2 cells were pretreated with PTL at different concentrations for 4 h and then incubated with NBD-cholesterol for 1 h. Values are expressed as mean ± SD of three to five experiments. (^#^: control, * *p* < 0.05, ** *p* < 0.01, *** *p* < 0.001, compared to control).

Caco-2 and HepG2 cells are common cell models for cholesterol absorption inhibition experiments [24,25,26] (Marques et al., 2018; Huang et al., 2021; Yao et al., 2020). In the present study, we examined whether PTL could prevent cholesterol absorption using these two cell models. NBD-cholesterol is a sensitive probe that binds cholesterol to NBD with fluorescent characteristics. Cholesterol uptake and efflux can be reflected in the fluorescence signal intensity. It has been applied to monitor intestinal cholesterol absorption in vivo [27] and is frequently used to create assays for cholesterol uptake by cells [28,29,30]. The NPC1L1 inhibitor ezetimibe, which can significantly inhibit cholesterol uptake, was employed as a positive control in the experiments. PTL had an inhibitory effect on cholesterol uptake in HepG2 cells, as shown in Table 1, and its IC_50_ value was around 50 μM. Figure 1A,C shows that PTL significantly and dose-dependently reduced cholesterol uptake in HepG2 and Caco-2 cells. PTL had virtually the same impact as ezetimibe at 50 μM in terms of lowering cholesterol uptake in Caco-2 cells (Figure 1A). Based on the findings above, PTL is capable of preventing cells from absorbing cholesterol.

### 2.2. Molecular Docking and Surface Plasmon Resonance (SPR)

**Figure 2:**
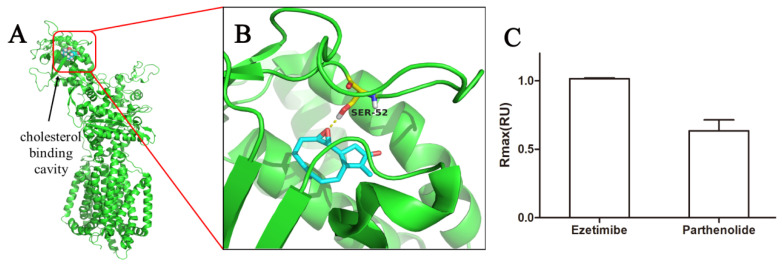
Binding modes of the PTL to NPC1L1. (**A**) The cholesterol-binding cavity (red) in the N-terminal domain. (**B**) Binding modes of the PTL to NPC1L1 (PDB: 6V3F). The PTL is shown in color by element (carbon in cyan). The H-bonds are displayed using yellow dashed lines. The key amino acid residues are shown in color by element (carbon in yellow). (**C**) Interaction assay of inhibitors with NPC1L1 by SPR. Data are presented as relative maximum binding in 50 μM. The binding of ezetimibe to NPC1L1 was normalized as 1.0.

According to the NBD-cholesterol results, PTL exhibited similar activity to ezetimibe and reduced cholesterol uptake in a concentration-dependent manner. NPC1L1 is a critical transporter for cellular cholesterol uptake so we speculated that PTL might inhibit cellular cholesterol uptake by interfering with NPC1L1. Later, we conducted molecular docking simulations at the N-terminus to see if PTL binds to NPC1L1, as the N-terminal domain of NPC1L1 can specifically bind cholesterol [31]. The docking results are displayed in Figure 2A,B. The findings demonstrated that PTL could bind to the N-terminal domain of NPC1L1’s cholesterol-binding site and that the interaction with PTL involved the amino acid residue Ser52. SPR is a label-free optical detection method that can be used to identify binding between two or more molecules quickly. As a result, we examined PTL’s binding to NPC1L1 using SPR (Figure 2C). The response to ezetimibe in NPC1L1 was normalized to 1.0. PTL demonstrated effective binding to NPC1L1. The observations of molecular docking and SPR, therefore, provisionally supported our hypothesis.

### 2.3. PTL Inhibits Cholesterol Uptake by Interacting with NPC1L1

**Figure 3:**
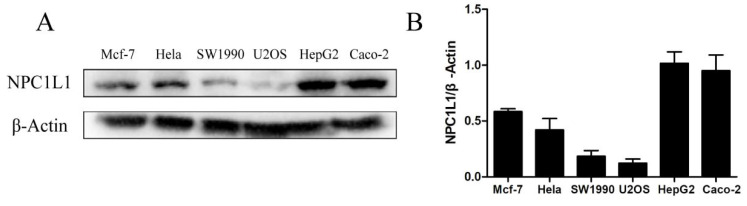
Expression of NPC1L1 in Mcf-7, Hela, SW1990, U2OS, HepG2, and Caco-2 cells. (**A**) Western blot analysis of cells after standard culture in medium A with 10% FBS. (**B**) A statistical representation of Western blot results.

Filipin is a fluorescent staining reagent that can specifically bind to cholesterol, form a complex, and generate fluorescence. The filipin staining method, employed in quantitative studies on intracellular cholesterol content, can be used to determine the amount of intracellular cholesterol present [32,33,34]. To identify potential cell models for the upcoming filipin experiment, we first assessed the protein expression of NPC1L1 in Mcf-7, Hela, SW1990, U2OS, HepG2, and Caco-2 cells. Western blot analysis revealed that NPC1L1 was highly expressed in Caco-2 and HepG2 cells, slightly more so in HepG2 than in Caco-2, and barely expressed in U2OS cells (Figure 3). NPC1L1 was also shown to be expressed in HepG2 and Caco-2 cells by Joanna P. Davies et al. [35] using mRNA and Western blot analysis, with HepG2 cells expressing more NPC1L1 protein than Caco-2 cells. These findings also support the use of HepG2 and Caco-2 cells to test PTL’s ability to prevent cholesterol absorption.

**Figure 4:**
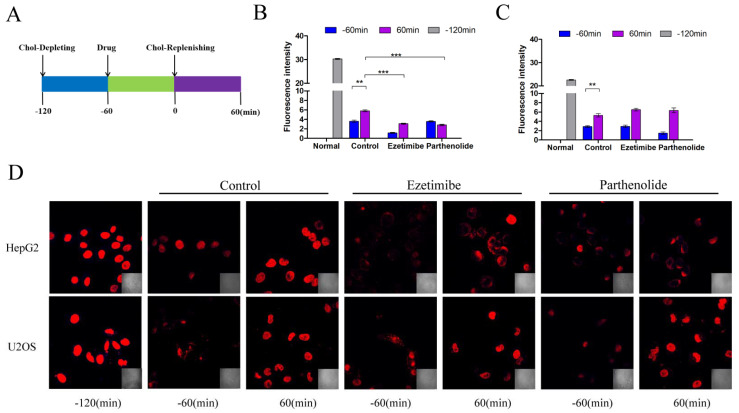
PTL inhibits cholesterol uptake. (**A**) Diagram showing the procedure used to treat the HepG2 and U2OS cells. Briefly, the cells were depleted of cholesterol by incubation in the cholesterol−depleting medium for 60 min. The cells were then incubated for 60 min in the cholesterol−depleted medium containing the indicated drugs. After 60 min, the cells were incubated for 60 min with the cholesterol−supplemented medium containing the indicated drugs. The PTL concentration was 50 μM and the ezetimibe concentration was 30 μM. Cells were subjected to staining at various time points. (**B**) Quantification of intracellular cholesterol of the HepG2 cells in (**D**). Error bars represent standard deviations. (**C**) Quantification of intracellular cholesterol of the U2OS cells in (**D**). Error bars represent standard deviations. (**D**) HepG2 and U2OS cells were treated as shown in (**A**). Cholesterol was labeled with filipin stain and examined using a high−resolution, high−speed scanning confocal microscope. Scale bar = 20 μm. (** *p* < 0.01, *** *p* < 0.001 compared to control).

According to the results of the Western blot, we chose HepG2 cells with high NPC1L1 expression and U2OS cells with low NPC1L1 expression in the subsequent filipin staining experiment. The method used was in reference to Liang Ge et al. [34]. Briefly, we added a medium that depletes cholesterol at −120 min and cultivated cells in that environment. The cholesterol-depleting medium was removed at 0 min and the cholesterol-replenishing medium was added. The cells were then grown for 1 h in the cholesterol-replenishing medium after the medication had been administered for 1 h (at −60 min) (Figure 4A). Subsequently, intracellular cholesterol was stained with filipin and evaluated using high-resolution, high-speed scanning confocal microscopy. We employed fat-free fetal bovine serum, lovastatin, and mevalonate in the formulation of the cholesterol-depleting medium, as per the procedure of Liang Ge et al. [34]. In this manner, intracellular cholesterol auto synthesis was suppressed, the impact of intracellular cholesterol auto synthesis on the outcomes of the experiments was diminished, and the cells received no additional supply of cholesterol. In the cholesterol-replenishing medium, the inclusion complex of cholesterol and CDX was included in addition to the ingredients listed above since CDX makes cholesterol more soluble.

In Figure 4, the experimental results are displayed. The fluorescence intensity shown in Figure 4D at −120 min (no treatment was given to HepG2 and U2OS cells at this time) corresponds to the cholesterol level in healthy cells. The fluorescence levels of the three groups of HepG2 and U2OS cells at −60 min decreased and were lower than at −120 min, indicating that the cells’ cholesterol content had decreased following the cholesterol-depleting culture. At 60 min, the intracellular fluorescence value increased and was higher than at −60 min but lower than at −120 min, indicating that the cells may not have had enough time to supplement cholesterol. It took longer for the cells to return to their normal cholesterol levels. In the control group, among others, the fluorescence dropped at −60 min and increased at 60 min, showing that the intracellular cholesterol decreased when the cholesterol was removed and increased after the cholesterol was supplemented, indicating that the cell modeling was successful. The same situation was also found in the ezetimibe and PTL groups. Ezetimibe was chosen as the promising medication because it targets the NPC1L1 protein [3]. In HepG2 cells with high NPC1L1 expression, the intracellular cholesterol content in the ezetimibe group was higher at 60 min than at −60 min but lower than in the control group, indicating that the increase in cholesterol in the ezetimibe group after cholesterol recovery was much lower than that in the control group. In addition, the membrane held the majority of the extra cholesterol. This may be due to the blocking of NPC1L1 by ezetimibe, thereby reducing cholesterol uptake by cells. The results in the PTL group were comparable and marginally superior to those in the ezetimibe group. When we quantitatively evaluated the intracellular cholesterol of HepG2 cells, we discovered that at 60 min, the control group was significantly different from the ezetimibe and the PTL groups (Figure 4B). However, in U2OS cells with low expression of NPC1L1, we found that after cholesterol supplementation, the fluorescence intensity of the ezetimibe and PTL groups was comparable to that of the control group, and there was no significant difference in the quantitative fluorescence analysis (Figure 4C). This demonstrated that PTL could only reduce cholesterol absorption when cells expressed NPC1L1, and when cells hardly expressed NPC1L1, it was unable to inhibit cholesterol uptake in the usual manner.

### 2.4. PTL Is a Competitive Inhibitor

**Figure 5:**
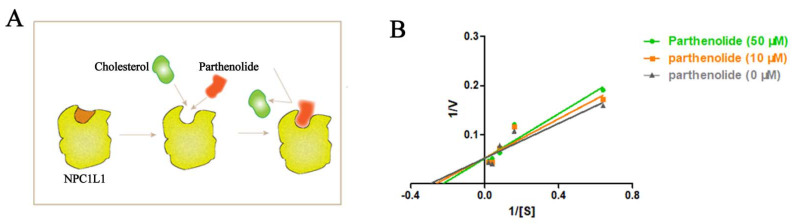
(**A**) Competitive inhibition mechanism of PTL. (**B**) Lineweaver−Burk plot in the presence of PTL at different concentrations.

The type of PTL-initiated NPC1L1 inhibition was established by kinetic studies. By using Lineweaver–Burk double-reciprocal plots, kinetic studies of PTL inhibition of cellular cholesterol uptake were conducted. The reaction rate and substrate concentration were used to determine whether PTL was a competitive inhibitor (Figure 5B). Figure 5A depicts our hypothesized PTL competitive inhibition mechanism in conjunction with the molecular docking results. PTL competed with NPC1L1 to bind to the same active site of cholesterol, amino acid residue Ser52, which interfered with the binding of NPC1L1 to cholesterol and reduced the binding rate of NPC1L1 to cholesterol. As a result of NPC1L1 being moved from the cytoplasm to the membrane, less cholesterol is transferred into the cytoplasm.

### 2.5. PTL Affects NPC1L1 Protein Expression in HepG2 Cells

**Figure 6:**
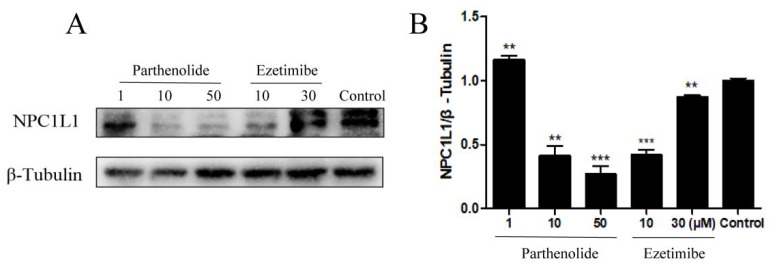
Effects of PTL on NPC1L1 protein expression in HepG2 cells. (**A**) Cells were treated with 1, 10, 50 μM of PTL and 10, 30 μM of ezetimibe for 24 h, followed by Western blot analysis of NPC1L1 in HepG2 cells. (**B**) A statistical representation of the Western blot showed that the expression of NPC1L1 was altered after PTL and ezetimibe treatments compared to controls. (** *p* < 0.01, *** *p* < 0.001 compared to control).

Intestinal cholesterol absorption was reduced by 70% in mice lacking the NPC1L1 gene, indicating that NPC1L1 is essential for increasing intestinal cholesterol absorption [3]. To determine if PTL could have an impact on NPC1L1 expression, we used the Western blot to quantify NPC1L1 in HepG2 cells (Figure 6A). Western blot analysis results revealed that the treatment of HepG2 cells with 50 μM PTL dramatically reduced the protein expression of NPC1L1 in a dose-dependent manner (Figure 6B). At a concentration of 10 μM, ezetimibe similarly decreased NPC1L1 protein expression. This revealed that PTL might reduce the cellular absorption of cholesterol by inhibiting NPC1L1’s function and decreasing its expression. Further investigation is required to determine how PTL alters the expression of NPC1L1.

### 2.6. PTL Has Little Cytotoxicity against Caco-2 and HepG2 Cells

**Figure 7:**
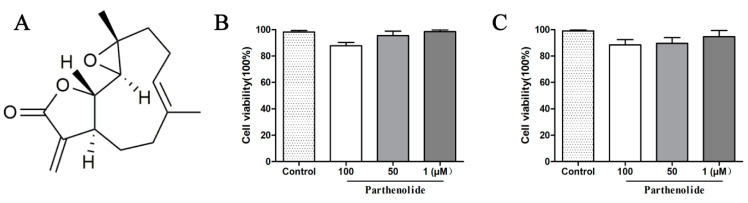
(**A**) Chemical structure of PTL. (**B**) Inhibition of Caco-2 cells’ and HepG2 cells’ viability by PTL at 100, 50, 1 μM. The experiments were performed in triplicate and are expressed as mean ± SD.

To examine the cytotoxicity of PTL, HepG2 cells and Caco-2 cells were exposed to concentrations of 100, 50, and 1 μM PTL for about 24 h, respectively. The vitality of the cells was then evaluated using the CCK-8 test. PTL at 100, 50, and 1 μM had little impact on the cell viability of HepG2 cells and Caco-2 compared to the control group (Figure 7B). The safety of PTL at the cellular level was preliminarily confirmed by our experimental findings, which can be applied to the thorough development of NPC1L1 inhibitors.

## 3. Discussion

Hypercholesterolemia is a significant risk factor for the development of arteriosclerotic cardiovascular disease. NPC1L1 is a crucial protein for the small intestine’s absorption of cholesterol and a potential target for medicines to decrease cholesterol [3]. Sesquiterpene lactones also exhibit lipid-lowering and anti-hyperlipidemic properties in addition to their anti-inflammatory properties [36,37,38]. PTL, a naturally occurring sesquiterpene lactone produced from Tanacetum parthenium, exhibits remarkable anti-inflammatory and anti-cancer properties [39,40,41], making it a key contender for future study and drug development [22]. This study is the first discussion of PTL’s potential ability to decrease cholesterol.

The balance between intestinal absorption and hepatic production of cholesterol determines the level of circulating plasma cholesterol [42]. Plasma cholesterol content and the rate of cholesterol absorption are intimately connected. We examined the impact of PTL on cholesterol absorption in Caco-2 and HepG2 cells since intestinal cholesterol absorption influences circulating cholesterol levels. We discovered that PTL prevented cellular uptake of cholesterol in a dose-dependent manner (Figure 1A,C). Our findings demonstrated that PTL suppressed cholesterol uptake by cells, with an IC_50_ value for cholesterol in HepG2 cells of 53.29 μM (Table 1). We speculated that PTL might lower cholesterol by a similar mechanism to ezetimibe, which involves reducing cellular uptake of cholesterol by acting on NPC1L1. This hypothesis was initially supported by molecular docking experiments, which demonstrated the connection between PTL and NPC1L1 (Figure 2A,B). Filipin staining tests were then carried out to fluorescently label cholesterol utilizing HepG2 cells with high NPC1L1 expression and U2OS cells with low NPC1L1 expression as cell models. The results also revealed that PTL’s action on NPC1L1 inhibited cells’ absorption of cholesterol (Figure 4). Subsequent kinetic studies confirmed that PTL inhibited NPC1L1’s ability to transport cholesterol in a competitive manner (Figure 5B). The additional benefit of lowering cholesterol absorption by inhibiting NPC1L1 is that it does not affect the absorption of nutrients requiring fat, such as fat-soluble triglycerides, vitamins, or bile acids [27]. Additionally, we discovered that PTL treatment dramatically decreased NPC1L1 protein levels in cells, and at 50 μM, the effect was superior to ezetimibe (Figure 6). Our findings imply that PTL can decrease cholesterol uptake in vitro and may be a possible NPC1L1 inhibitor. These results could increase the range of alternatives for treating hypercholesterolemia. In our group, additional research on the potential use of PTL in the management of hypercholesterolemia is ongoing.

## 4. Materials and Methods

### 4.1. Materials

The HepG2 cell line, the Caco-2 cell line, filipin, Dulbecco’s modified Eagle’s medium, penicillin/streptomycin (batch number PWL062), and the BCA protein quantification kit (batch number MA0082-2) were purchased from Meilun Bio., Dalian, China; fetal bovine serum (FBS) was purchased from Gibco, Billings, MT, USA; dimethyl sulfoxide (DMSO, lot number D8371) and the cell viability CCK-8 kit (lot number MA0718-L) were purchased from the Biyuntian Institute of Biotechnology, Shanghai, China. NBD-cholesterol was purchased from Bailingwei, Beijing, China; PTL, mevalonate, and methyl-β-cyclodextrin (CDX) were purchased from Sigma, New York, NY, USA; lovastatin and cholesterol were purchased from Macklin, Shanghai, China; defatted fetal bovine serum (LPDS) was purchased from Biological Industries, Israel.

### 4.2. Cell Culture

HepG2 (human hepatocellular carcinomas), Caco-2 (human epithelial colorectal adenocarcinoma cell line), MCF-7 (human breast cancer cell line), Hela (Human cervical cancer cell line), SW1990 (human pancreatic cancer cell line) and U2OS (human osteosarcoma cell line) cells were grown in the incubator at 37 °C in 5% CO_2_. The cells were maintained in medium A (Dulbecco’s modified Eagle’s medium containing 100 units/mL penicillin and 100 mg/mL streptomycin sulfate) supplemented with 10% FBS. Cholesterol-depleting medium was medium A supplemented with 5% LPDS, 10 μM lovastatin, 50 μM mevalonate, and 1.5% CDX. Cholesterol-replenishing medium contained medium A supplemented with 5% LPDS, 10 μM lovastatin, 50 μM mevalonate, and 15 μg/mL of cholesterol/CDX. The sterol/CDX inclusion complexes were prepared as described previously [43].

### 4.3. CCK-8 Cell Viability Assay

HepG2 and Caco-2 cells were cultured in medium A containing 10% FBS. Cells in the logarithmic growth phase were adjusted to 3 × 10^5^ cells/mL, and 100 μL of cell suspension was plated in a 96-well plate and cultured at 37 °C for 24 h. Then, 10 μL of PTL (100 μM) was added to each well. After incubation for 24 h, 10 μL of CCK-8 solution was pipetted into the 96-well plate and incubated for 1 h. The absorbance at 450 nm was measured with a microplate reader.

### 4.4. Cholesterol Uptake Assay

The fluorescent analog, 22-(N-(7-Nitrobenz-2-Oxa-1,3-Diazol-4-yl) Amino)-23,24-Bisnor-5-Cholen-3β-Ol (NBD-cholesterol), has previously been demonstrated to trace cholesterol absorption in vitro and in vivo and is, therefore, a good tool for exploring cholesterol uptake using a non-radioactive fluorescent alternative [27]. To simulate the intestinal environment, drugs and NBD-cholesterol were formulated using 0.5 mM taurine sodium salt (Beyotime, Shanghai, China) and medium A containing LPDS. Before checking cholesterol absorption, the culture medium with 10% FBS was replaced with the medium containing the LPDS, followed by incubating the cells for 24 h. Cells were then washed 3 times with PBS buffer, incubated with drugs for 4 h, and then NBD-cholesterol for 1 h. Cells were washed three times in PBS to remove any NBD-cholesterol that was not associated with the cells, and then the fluorescence was measured at excitation and emission wavelengths of λex = 485 nm and λem = 535 nm, respectively.

### 4.5. Filipin Staining

Filipin (25 mg/mL in DMSO) was diluted in PBS/10% FBS at a concentration of 0.05 mg/mL as a working solution. Cells were cultured in confocal culture chambers. After washing three times with PBS, cells were fixed in 4% paraformaldehyde for 20 min at room temperature, washed three times with PBS, stained with filipin working solution for 2 h at room temperature, and then washed three times with PBS. Images were obtained by observing cells with a high-resolution, high-speed scanning confocal microscope (Nikon AIR HD25, Tokyo, Japan).

### 4.6. Western Blot

The total cellular proteins were prepared using RIPA buffer (Meilun Biotechnology, Dalian, China). The protein lysates were analyzed by 8% sodium dodecyl sulfate-polyacrylamide gel electrophoresis and then transferred onto a PVDF membrane (Millipore Corporation, Darmstadt, Germany). Then, the NPC1L1 antibody (1:1000, CST, Parsons, KS, USA) was used as the primary antibody to incubate overnight at 4 °C. Then, the membrane was incubated with a horseradish peroxidase-conjugated secondary antibody (1:2000, rabbit; ABclonal, Wuhan, China) for 1 h at room temperature. β-tubulin and β-Actin were used as controls. Finally, the PVDF membrane was visualized using an enhanced chemiluminescence (ECL) kit (Meilun Biotechnology, China). All experiments were evaluated by densitometric analysis with ImageJ software (National Institutes of Health, Bethesda, MD, USA).

### 4.7. Kinetic Analysis

To analyze the dynamics of molecules, 3 × 10^3^ HepG2 cells were evenly seeded in a 96-well plate, cultured in medium A with 10% FBS, and placed in an incubator at 37 °C and 5% CO_2_ for 24 h. After 24 h, cells were washed 2–3 times with ice-cold 4 °C PBS. Medium A was added and incubated at 37 °C under a 5% CO_2_ atmosphere. After 24 h, different concentrations of PTL (50 μM,10 μM, 0 μM) were added and incubated for 4 h. Then various concentrations of NBD-cholesterol (50 μM, 25 μM, 12.5 μM, 6.25 μM, and 1.5625 μM) were added and incubated for 1 h. Then, cells were washed 2–3 times with ice-cold 4 °C PBS and the fluorescence density was measured at an excitation wavelength of 485 nm and an emission wavelength of 535 nm. The Lineweaver–Burk double reciprocal plot was used to determine the type of inhibition. All the analyses were carried out in triplicate and the mean ± standard deviation values were used to plot the graph.

### 4.8. Method of Molecular Docking

The experiment was performed as previously described [44]. Briefly, docking simulations were performed with SYBYL-X 2.0 software. All ligand molecules were plotted using standard parameters for SYBYL-X. Their geometric conformational energy was minimized for 1000 steps using the Tripos force field, and Gasteiger–Huckel charges were calculated. Protein receptors were prepared using standard methods. Hydrogen bonds were indicated by dashed lines. Pymol was used to observe whether the ligands and protein receptors interacted.

### 4.9. SPR Studies

SPR interaction analysis was conducted using Biacore T200 from GE Healthcare Life Sciences. CM5 series sensor chips and coupling reagents EDC and NHS were purchased from GE. NPC1L1 (final concentration 50 μg/mL) was dissolved in 10 mM sodium acetate buffer (pH 4.4) and immobilized on a CM5 chip. The surface of the flow cell was activated for 7 min using a 1:1 mixture of 100 mM EDC and 100 mM NHS at a flow rate of 10 μL/min. Subsequently, the corresponding proteins were injected over the surface for 7 min using a time and flow method (final immobilization level: 15,000 RU). Excess reactive esters on the sensor chip surface were blocked with 1 M ethanolamine (pH 8.5) for 7 min. The flow cell used for reference was activated and blocked as described above but remained uncoupled. Binding was usually reported in response units (RU), defined as the response obtained from the flow cell containing the immobilized receptor minus the response obtained from the reference flow cell.

### 4.10. Statistical Analysis

The data were expressed as mean ± SD and analyzed with GraphPad Prism 5.0. The differences among multiple groups were examined using one-way ANOVA. *p* values < 0.05 were considered statistically significant.

## Figures and Tables

**Table 1:** IC_50_ value of PTL for inhibition of cholesterol uptake.

Inhibitors	PTL	Ezetimibe
IC_50_ (μM)	53.29 ± 1.39	51.49 ± 0.6652

## Data Availability

Not applicable.

## Abbreviations

PTL: parthenolide; NPC1L1: Niemann–Pick C1-like 1; HMG-CoA reductase: 3-hydroxy-3-methylglutaryl-CoA reductase; LDL: low-density lipoprotein; PCSK9: proprotein convertase subtilisin/kexin type 9; SPR: Surface plasmon resonance; FBS: fetal bovine serum; CDX: methyl-β-cyclodextrin; LPDS: defatted fetal bovine serum; NBD-cholesterol: 22-(N-(7-Nitrobenz-2-Oxa-1,3-Diazol-4-yl) Amino)-23,24-Bisnor-5-Cholen-3β-Ol; RU: response units; DMSO: dimethyl sulfoxide.

## References

[1] Mirzadeh A.F., Arabian M., Maleki M., Malakootian M. (2020). Small Molecules with Big Impacts on Cardiovascular Diseases. Biochem. Genet..

[2] Huff M.W., Burnett J.R. (1997). 3-Hydroxy-3-methylglutaryl coenzyme a reductase inhibitors and hepatic apolipoprotein B secretion. Curr. Opin. Lipidol..

[3] Altmann S.W., Davis H.J., Zhu L.J., Yao X., Hoos L.M., Tetzloff G., Iyer S.P., Maguire M., Golovko A., Zeng M. (2004). Niemann-Pick C1 Like 1 protein is critical for intestinal cholesterol absorption. Science.

[4] Zhang R., Liu W., Zeng J., Meng J., Jiang H., Wang J., Xing D. (2022). Niemann-Pick C1-Like 1 inhibitors for reducing cholesterol absorption. Eur. J. Med. Chem..

[5] Dias C.S., Shaywitz A.J., Wasserman S.M., Smith B.P., Gao B., Stolman D.S., Crispino C.P., Smirnakis K.V., Emery M.G., Colbert A. (2012). Effects of AMG 145 on low-density lipoprotein cholesterol levels: Results from 2 randomized, double-blind, placebo-controlled, ascending-dose phase 1 studies in healthy volunteers and hypercholesterolemic subjects on statins. J. Am. Coll. Cardiol..

[6] Xu Q., Deng Y., Xiao J., Liu X., Zhou M., Ren Z., Peng J., Tang Y., Jiang Z., Tang Z. (2021). Three musketeers for lowering cholesterol: Statins, ezetimibe and evolocumab. Curr. Med. Chem..

[7] Chalasani N., Aljadhey H., Kesterson J., Murray M.D., Hall S.D. (2004). Patients with elevated liver enzymes are not at higher risk for statin hepatotoxicity. Gastroenterology.

[8] Xu F., Chen J., Zhang Y., Wu Q., Shen Y., Gu W., Liu S., Lu C., Liao H., Bao K. (2020). Molecular insight into the mechanism of lipid regulating effect of Alisma orientalis based on ACAT. Int. J. Biol. Macromol..

[9] Bahmani M., Mirhoseini M., Shirzad H., Sedighi M., Shahinfard N., Rafieian-Kopaei M. (2015). A review on promising natural agents effective on hyperlipidemia. J. Evid. Based Complementary Altern. Med..

[10] Wiviott S.D., Cannon C.P. (2006). Update on lipid-lowering therapy and LDL-cholesterol targets. Nat. Clin. Pract. Cardiovasc. Med..

[11] Wiedhopf R.M., Young M., Bianchi E., Cole J.R. (1973). Tumor inhibitory agent from Magnolia grandiflora (Magnoliaceae). I: Parthenolide. J. Pharm. Sci..

[12] Zhang S., Ong C.N., Shen H.M. (2004). Critical roles of intracellular thiols and calcium in parthenolide-induced apoptosis in human colorectal cancer cells. Cancer Lett..

[13] D’Anneo A., Carlisi D., Lauricella M., Emanuele S., Di Fiore R., Vento R., Tesoriere G. (2013). Parthenolide induces caspase-independent and AIF-mediated cell death in human osteosarcoma and melanoma cells. J. Cell. Physiol..

[14] Liu J.W., Cai M.X., Xin Y., Wu Q.S., Ma J., Yang P., Xie H.Y., Huang D.S. (2010). Parthenolide induces proliferation inhibition and apoptosis of pancreatic cancer cells In Vitro. J. Exp. Clin. Cancer Res..

[15] Sun Y., St C.D., Xu Y., Crooks P.A., St C.W. (2010). A NADPH oxidase-dependent redox signaling pathway mediates the selective radiosensitization effect of parthenolide in prostate cancer cells. Cancer Res..

[16] Jeyamohan S., Moorthy R.K., Kannan M.K., Arockiam A.J. (2016). Parthenolide induces apoptosis and autophagy through the suppression of PI3K/Akt signaling pathway in cervical cancer. Biotechnol. Lett..

[17] Liu D., Han Y., Liu L., Ren X., Zhang H., Fan S., Qin T., Li L. (2021). Parthenolide inhibits the tumor characteristics of renal cell carcinoma. Int. J. Oncol..

[18] Heinrich M., Robles M., West J.E., Ortiz D.M.B., Rodriguez E. (1998). Ethnopharmacology of Mexican asteraceae (Compositae). Annu. Rev Pharm. Toxicol..

[19] Saadane A., Masters S., Didonato J., Li J., Berger M. (2007). Parthenolide inhibits IkappaB kinase, NF-kappaB activation, and inflammatory response in cystic fibrosis cells and mice. Am. J. Respir. Cell Mol. Biol..

[20] Hehner S.P., Heinrich M., Bork P.M., Vogt M., Ratter F., Lehmann V., Schulze-Osthoff K., Droge W., Schmitz M.L. (1998). Sesquiterpene lactones specifically inhibit activation of NF-kappa B by preventing the degradation of I kappa B-alpha and I kappa B-beta. J. Biol. Chem..

[21] Guzman M.L., Rossi R.M., Karnischky L., Li X., Peterson D.R., Howard D.S., Jordan C.T. (2005). The sesquiterpene lactone parthenolide induces apoptosis of human acute myelogenous leukemia stem and progenitor cells. Blood.

[22] Mathema V.B., Koh Y.S., Thakuri B.C., Sillanpaa M. (2012). Parthenolide, a sesquiterpene lactone, expresses multiple anti-cancer and anti-inflammatory activities. Inflammation.

[23] Kim C.Y., Kang B., Hong J., Choi H.S. (2020). Parthenolide inhibits lipid accumulation via activation of Nrf2/Keap1 signaling during adipocyte differentiation. Food Sci. Biotechnol..

[24] Marques M.R., Cerda A., Fontanari G.G., Pimenta D.C., Soares-Freitas R.M., Hirata M.H., Hirata R., Areas J. (2018). Transport of cowpea bean derived peptides and their modulator effects on mRNA expression of cholesterol-related genes in Caco-2 and HepG2 cells. Food Res. Int..

[25] Huang Y., Tocmo R., Nauman M.C., Haughan M.A., Johnson J.J. (2021). Defining the cholesterol lowering mechanism of bergamot (Citrus bergamia) extract in HepG2 and caco-2 cells. Nutrients.

[26] Yao Y., Xu F., Ju X., Li Z., Wang L. (2020). Lipid-Lowering effects and intestinal transport of polyphenol extract from digested buckwheat in Caco-2/HepG2 coculture models. J. Agric. Food Chem..

[27] Sparrow C.P., Patel S., Baffic J., Chao Y.S., Hernandez M., Lam M.H., Montenegro J., Wright S.D., Detmers P.A. (1999). A fluorescent cholesterol analog traces cholesterol absorption in hamsters and is esterified in vivo and In Vitro. J. Lipid Res..

[28] Thilavech T., Adisakwattana S. (2019). Cyanidin-3-rutinoside acts as a natural inhibitor of intestinal lipid digestion and absorption. BMC Complement. Altern. Med..

[29] Yao S.L., Xu Y., Zhang Y.Y., Lu Y.H. (2013). Black rice and anthocyanins induce inhibition of cholesterol absorption in vitro. Food Funct..

[30] Fuentes M., Santander N., Cortes V. (2018). Insulin increases cholesterol uptake, lipid droplet content, and apolipoprotein B secretion in CaCo-2 cells by upregulating SR-BI via a PI3K, AKT, and mTOR-dependent pathway. J. Cell. Biochem..

[31] Zhang J.H., Ge L., Qi W., Zhang L., Miao H.H., Li B.L., Yang M., Song B.L. (2011). The N-terminal domain of NPC1L1 protein binds cholesterol and plays essential roles in cholesterol uptake. J. Biol. Chem..

[32] Bornig H., Geyer G. (1974). Staining of cholesterol with the fluorescent antibiotic “filipin”. Acta Histochem..

[33] Kruth H.S., Fry D.L. (1984). Histochemical detection and differentiation of free and esterified cholesterol in swine atherosclerosis using filipin. Exp. Mol. Pathol..

[34] Ge L., Wang J., Qi W., Miao H.H., Cao J., Qu Y.X., Li B.L., Song B.L. (2008). The cholesterol absorption inhibitor ezetimibe acts by blocking the sterol-induced internalization of NPC1L1. Cell Metab..

[35] Davies J.P., Scott C., Oishi K., Liapis A., Ioannou Y.A. (2005). Inactivation of NPC1L1 causes multiple lipid transport defects and protects against diet-induced hypercholesterolemia. J. Biol. Chem..

[36] Wang X., Liu M., Cai G.H., Chen Y., Shi X.C., Zhang C.C., Xia B., Xie B.C., Liu H., Zhang R.X. (2020). A potential nutraceutical candidate lactucin inhibits adipogenesis through downregulation of JAK2/STAT3 signaling Pathway-Mediated mitotic clonal expansion. Cells.

[37] Asgary S., Naderi G.H., Sarrafzadegan N., Mohammadifard N., Mostafavi S., Vakili R. (2000). Antihypertensive and antihyperlipidemic effects of Achillea wilhelmsii. Drugs Exp. Clin. Res..

[38] Hall I.H., Lee K.H., Starnes C.O., Muraoka O., Sumida Y., Waddell T.G. (1980). Antihyperlipidemic activity of sesquiterpene lactones and related compounds. J. Pharm. Sci..

[39] Kim S.L., Kim S.H., Trang K.T., Kim I.H., Lee S.O., Lee S.T., Kim D.G., Kang S.B., Kim S.W. (2013). Synergistic antitumor effect of 5-fluorouracil in combination with parthenolide in human colorectal cancer. Cancer Lett..

[40] Liu Y.J., Tang B., Wang F.C., Tang L., Lei Y.Y., Luo Y., Huang S.J., Yang M., Wu L.Y., Wang W. (2020). Parthenolide ameliorates colon inflammation through regulating Treg/Th17 balance in a gut microbiota-dependent manner. Theranostics.

[41] Freund R., Gobrecht P., Fischer D., Arndt H.D. (2020). Advances in chemistry and bioactivity of parthenolide. Nat. Prod. Rep..

[42] Park J.G., Oh G.T. (2011). The role of peroxidases in the pathogenesis of atherosclerosis. BMB Rep..

[43] Brown A.J., Sun L., Feramisco J.D., Brown M.S., Goldstein J.L. (2002). Cholesterol addition to ER membranes alters conformation of SCAP, the SREBP escort protein that regulates cholesterol metabolism. Mol. Cell.

[44] Zhang R., Song Z., Wang X., Xue J., Xing D. (2021). One-step modification to identify dual-inhibitors targeting both pancreatic triglyceride lipase and Niemann-Pick C1-like 1. Eur. J. Med. Chem..

